# Effect of Carbon Nanostructures and Fatty Acid Treatment on the Mechanical and Thermal Performances of Flax/Polypropylene Composites

**DOI:** 10.3390/polym12020438

**Published:** 2020-02-13

**Authors:** Pietro Russo, Libera Vitiello, Francesca Sbardella, Jose I. Santos, Jacopo Tirillò, Maria Paola Bracciale, Iván Rivilla, Fabrizio Sarasini

**Affiliations:** 1 Institute for Polymers, Composites and Biomaterials, National Council of Research, 80078 Pozzuoli, Italy; pietro.russo@ipcb.cnr.it (P.R.); liberavitiello29@gmail.com (L.V.); 2 Department of Chemical Engineering Materials Environment & UdR INSTM, Sapienza-Università di Roma, 00184 Roma, Italy; jacopo.tirillo@uniroma1.it (J.T.); mariapaola.bracciale@uniroma1.it (M.P.B.); ivan.rivilla@ehu.es (I.R.); 3 SGIker-UPV/EHU, Centro “Joxe Mari Korta”, Tolosa Hiribidea, 72, 20018 Donostia−San Sebastián, Spain; joseignacio.santosg@ehu.es; 4 Donostia International Physics Center, 20018 Donostia-San Sebastián, Spain

**Keywords:** polymer matrix composites, flax fibers, surface treatments, adhesion

## Abstract

Four different strategies for mitigating the highly hydrophilic nature of flax fibers were investigated with a view to increase their compatibility with apolar polypropylene. The effects of two carbon nanostructures (graphene nanoplatelets (GNPs) and carbon nanotubes (CNTs)), of a chemical modification with a fatty acid (stearic acid), and of maleated polypropylene on interfacial adhesion, mechanical properties (tensile and flexural), and thermal stability (TGA) were compared. The best performance was achieved by a synergistic combination of GNPs and maleated polypropylene, which resulted in an increase in tensile strength and modulus of 42.46% and 54.96%, respectively, compared to baseline composites. Stearation proved to be an effective strategy for increasing the compatibility with apolar matrices when performed in an ethanol solution with a 0.4 M concentration. The results demonstrate that an adequate selection of surface modification strategies leads to considerable enhancements in targeted properties.

## 1. Introduction

Natural-fiber-reinforced composites have received attention over the recent years because of their potential ability to replace their synthetic counterparts in an attempt to meet the new regulations that promote the use of more sustainable and recyclable materials [1,2]. The high specific mechanical properties and the carbon dioxide neutrality of natural fibers have already stimulated the replacement of glass fibers in several sectors, especially the automotive and construction ones, but usually as secondary load-bearing structures [3,4].

A step forward is their use in structural applications, but some challenges still need to be properly faced and solved [5]. The variability in physical and mechanical properties, due to their natural origin, is difficult to manage unless the fiber supply chain is carefully controlled and the manufacturing processes are optimized [6]. Goudenhooft et al. [7] recently showed that tensile properties of flax fibers are not significantly affected over time, regardless of the fiber yield and variety, and that the resulting dispersion in the specific mechanical properties is in the same range as that of glass fibers. Another significant issue is related to the processing conditions of the composites (temperature, dwell time, pressure), which have a major impact on the final mechanical properties, especially for thermoplastic-based composites [8,9,10].

The last topic of considerable interest is the extent of fiber/matrix interfacial adhesion. It is well known that the mechanical properties of composites are dictated not only by the inherent properties of the constituents, but also by the fiber/matrix interface. The poor compatibility with polymer matrices (especially thermoplastics) due to their hydrophilic behavior still represents a major limitation for a wider industrial exploitation of natural fibers [11]. Several efforts to enhance the interfacial adhesion of natural fibers have been proposed, including chemical [12,13,14,15,16] and physical treatments [17,18,19,20], but their industrial implementations are often complicated by the large amounts of chemicals involved or the multiple processing steps required. A more recent approach deals with the grafting of nanostructures onto fiber surfaces to increase the adhesion with the polymer matrix. This strategy has been widely exploited for synthetic fibers, such as glass [21,22,23] and carbon fibers [24,25,26], but has attracted less attention in the field of natural fibers. Wang et al. [27] modified the surfaces of flax fibers by grafting TiO_2_ nanoparticles using a silane coupling agent. The authors reported an increase in tensile strength and interfacial strength with an epoxy matrix of 23.1% and 40.5%, respectively. Copper nanoparticles on flax fibers were found to produce significant improvements in fiber tensile modulus and strength, equal to 50% and 75%, respectively [28]. Ajith et al. [29] modified flax yarns with hydrous zirconia nanoparticles synthesized by hydrolysis of a zirconium oxychloride solution. The presence of these nanoparticles resulted in an increase in single fiber tensile strength and interfacial strength with an epoxy matrix of 85% and 65%, respectively. Lakshmanan and Chakraborty [30] synthesized and deposited silver nanoparticles on jute fibers without deteriorating the mechanical properties of the fibers. In addition, the modified fabrics exhibited good antibacterial properties. In [31], the authors reported a simple spray-coating process to deposit carbon nanotubes (CNTs) over the surfaces of ramie fibers. This coating enhanced the flexural strength and modulus of an epoxy-based composite by 38.4% and 36.8%, respectively, while a microdebonding test highlighted an increase in the interfacial shear strength of 25.7%. Sarker et al. [32] coated graphene materials, i.e., graphene oxide (GO) and graphene flakes (G), on alkali-treated jute fibers, and an interfacial shear strength enhancement of ~236% compared to untreated fibers was achieved. In [33], the authors coupled a jute fiber individualization procedure with the grafting of GO and subsequent hot pressing to get preforms that were then vacuum-infused with epoxy matrix. The graphene coating resulted in a dramatic increase in tensile modulus and strength of the jute–epoxy composites compared to untreated composites of 324% and 110%, respectively. Grafting of nanometer-sized materials can therefore be considered as an effective method for improving fiber/matrix interfacial adhesion, thus leading to the manufacturing of high-performance natural-fiber-reinforced composites. Another positive feature of this strategy is the possibility of adding functionalities to the resulting composites. Zhuang et al. [34] deposited multi-walled carbon nanotubes (MWCNTs) on the surfaces of jute fibers, and the epoxy-based composites exhibited multifunctional sensing abilities for temperature, moisture, and strain. In [35], graphene nanoplatelets (GNPs) and carbon black were used to make flax yarns electrically conductive; these were then used to fabricate stretchable strain sensors with gauge factors ranging from 1.46 up to 5.62, and a reliability for sensing strains of up to 60%.

The need to optimize the interfacial adhesion in natural-fiber composites is even more important with thermoplastic-based composites due to the non-polar nature of most of them. In particular, polypropylene (PP) is one of the most widely used polyolefins. Its low density, low price, good mechanical properties, good processability, and recyclability make it a popular material as a matrix for natural fiber composites [10,36,37]. Flax fibers currently account for about half of the natural fibers used in automotive applications, followed by kenaf and hemp [4], and the combination of PP/flax has been widely investigated in literature, highlighting the dramatic incompatibility between these two constituents. In an attempt to tailor the properties of natural fibers for their subsequent successful application in high-performance plant-fiber composites but with limited costs and environmental impact, in this work, we investigated the interfacial interactions in flax/PP composites through two different approaches: (i) The grafting of carbon nanostructures (CNTs and GNPs) and (ii) the chemical modification with a fatty acid (stearic acid). In both cases, the addition of a maleic-anhydride-modified polypropylene (MAPP) was also used to tune the interfacial adhesion. In particular, stearic acid, a long alkyl chain fatty acid, was used to lower the hydrophilic character of flax fibers. This surface modification treatment has already been used with limited success in other studies [38,39,40,41], even though a detailed investigation on the effects of its concentration on the surface properties of flax fibers has not been reported so far. In addition, grafting of nanostructures for improving interfacial adhesion has mostly been exploited for thermoset-based composites, and scarcely with thermoplastic polymers [42]. The morphology and the thermal stability of flax/PP composites were characterized, and the impacts of the surface treatments or compatibilization with MAPP on their mechanical performance were addressed.

## 2. Materials and Methods

### 2.1. Materials 

The composites investigated in this study are based on a polypropylene (PP) matrix (Hyosung Topilene PP J640, MFI@230 °C,2.16 kg: 10 g/10 min) supplied by Songhan Plastic Technology Co. Ltd. (Shanghai City, China) and a commercial 2 × 2 twill flax fabric (areal weight: 200 g/m^2^) commercialized without any specific sizing agent and supplied by Composites Evolution (Chesterfield, UK). The matrix was used as received or pre-modified by inclusion of 2 wt.% of a coupling agent, Polybond 3000 (maleic-anhydride-modified PP, MFI@190 °C, 2.16 kg: 400 g/10 min) from Chemtura (Cologne, Germany). The stearic acid (SA), ethanol, and toluene were of analytical grade and used without further purifications. Carbon nanotubes (CNTs) with average length < 1 μm, average outer diameter < 9.5 nm, and bulk density of 100 g/L were provided by Nanocyl SA (Sambreville, Belgium) with the code NC3150. The graphene nanoplatelets (GNPs) supplied by Nanesa s.r.l. (Arezzo, Italy) in the form of black powder have an average flake thickness of 40 nm corresponding to 40 stacked layers, an average particle size of 30 μm, a bulk density of 20–42 g/L, and a specific surface area (BET) > 30 m^2^/g.

### 2.2. Surface Treatment of Flax Fabrics with Carbon Nanostructures 

The adopted procedure involved the preparation of an aqueous dispersion of the carbonaceous filler with a concentration equal to 0.5 wt.%. In the case of carbon nanotubes, the dispersion was performed in the presence of 1% by weight of a Triton X-100 surfactant supplied by Sigma Aldrich (Milano, Italy). Layers of flax fabric, already cut to such dimensions as to be used for the preparation of the laminated samples, were immersed for 30 min at room temperature in these dispersions and pre-sonicated for 180 min at room temperature. Finally, the wet fabric layers were subjected to drying for 30 min at 80 °C in a ventilated oven.

### 2.3. Surface Treatment of Flax Fabrics with Stearic Acid 

**Scheme 1:**
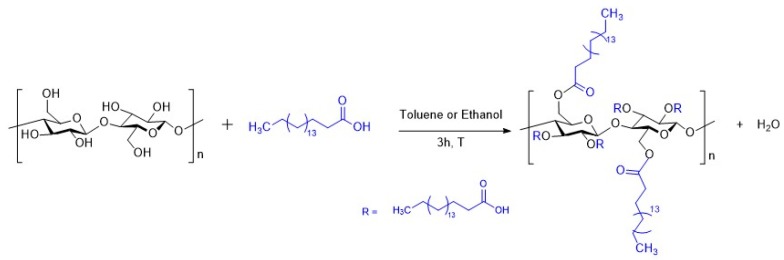
Synthetic route for the modification of the flax fabric with stearic acid (SA).

Different concentrations (0.1, 0.2, 0.3, and 0.4 M) of stearic acid in toluene or ethanol were prepared. These solutions were heated at temperatures close to the boiling points of the solvents, 100 °C for toluene and 65 °C for ethanol, respectively. Once this temperature was reached, the reaction mixture, including the flax fabric, was maintained for 3 h and then washed three times with deionized water and dried at room temperature (Scheme 1). The carboxyl group (–COOH) is supposed to react with the hydroxyl groups of the fiber through an esterification reaction and, hence, the treatment should reduce the number of hydroxyl groups available for bonding with water molecules. Furthermore, the long hydrocarbon chain of stearic acid (18 carbon atoms) provides an extra protection from water due to its hydrophobic nature [43].

### 2.4. Composite Manufacturing 

Composite samples with a symmetrical stacking sequence [0/90] and consisting of 8 plies were obtained by alternately stacking polymer films and layers of as-received or pre-treated flax fabrics, pre-conditioned in a vacuum oven at 70 °C for 2 h, and subsequently underwent hot compression at 210 °C. This last step was carried out using a Collin GmbH (Edersberg, Germany) model P400E press according to a pre-optimized pressure cycle: 2 min—0 bar, 2 min—5 bar, 2 min—15 bar, 1 min—25 bar, 2 min—35 bar, 2 min—40 bar. Finally, the cooling of the composite plates to 30 °C was conducted at a constant pressure of 40 bar before releasing the pressure and extracting the sample. These process conditions provided the laminates with an average thickness of 2.5 mm and with a fiber content of approximately 45% by volume. Films of PP and PPC (PP modified with coupling agent) with a thickness approximately equal to 80 μm were obtained with a Collin Teach-Line E 20-T single-head extruder and Collin CR 72T calender (Ebersberg, Germany), setting a screw speed of 55 rpm and a temperature profile from the hopper to the die equal to: 180–190–200–190–185 °C.

### 2.5. Characterization Techniques 

The mechanical properties of the flax fabrics before and after surface modification treatments were assessed according to ASTM D5035. Tensile tests were carried out at room temperature by means of a Zwick/Roell Z010 (Ulm, Germany) equipped with a 10 kN load cell. A gauge length of 75 mm was used for specimens with a width equal to 25 mm. Tests were performed in displacement control at a crosshead speed of 100 mm/min to ensure failure within 20 ± 3 s. At least five tests were performed for each fabric.

The mechanical properties of the composites were investigated in quasi-static tensile and flexural tests with a Zwick/Roell Z010. For tensile measurements, a gauge length of 60 mm and a cross-head speed of 5 mm/min were set in accordance with ASTM D3039, while flexural tests were conducted in a three-point bending configuration with a span of 76 mm and a speed of loading equal to 5 mm/min as per ASTM D790. Five tests were carried out for each composite formulation.

FT-IR spectra were carried out with a Bruker Vertex 70 spectrometer (Bruker Optik GmbH, Ettlingen, Germany) equipped with a single reflection Diamond ATR (Attenuated Total Reflectance) cell. The ATR-FTIR spectrum was recorded with a 3 cm^−1^ spectral resolution in the mid-infrared range (350–4000 cm^−1^) using 256 scans.

CPMAS (Cross Polarization/Magic Angle Spinning) NMR spectra were recorded on a 9.4T (400 MHz) Bruker (Billerica, USA) system equipped with a 4 mm MASDVT Double Resonance HX MAS probe. Larmor frequencies were 400.17 MHz and 100.63 MHz for ^1^H and ^13^C nuclei, respectively. Chemical shifts were calibrated indirectly with glycin, with a carbonyl peak at 176 ppm. The sample rotation frequency was 10 kHz and the relaxation delay was 5 s. The number of scans was 4096. Polarization transfer was achieved with RAMP cross-polarization (ramp on the proton channel) with a contact time of 5 ms. High-power SPINAL 64 heteronuclear proton decoupling was applied during acquisition.

X-ray diffraction (XRD) analysis was performed with a diffractometer X’Pert PRO by Philips (Malvern, UK) (CuKα radiation = 1.54060 Å; 40 kV and 40 mA) at room temperature. XRD patterns were collected in the range of 2θ = 10°–80° with a step size of 0.02° scan and a time per step of 3 s.

Surface wettability tests were performed measuring the contact angles of water droplets on the twill fabric surface using an optical analyzer (OCA15Pro, DataPhysics Instruments, Filderstadt, Germany). The static sessile method with a droplet volume of 3 μL was selected and a Milli-Q ultrapure water was used as the testing liquid. A minimum of ten droplets localized on different areas of the flax fabric samples were analyzed. Contact angle values were determined by drop shape analysis using the DataPhysics SCA 20 software module.

A scanning electron microscope, FEG Mira3 (Tescan, Brno, Czech Republic), was used to analyze the morphologies of neat and pre-treated flax fabrics as well as those of fractured surfaces of composite laminates. Prior to observation, the fracture surfaces were sputter-coated with gold.

## 3. Results and Discussion

### 3.1. Characterization of Composites Reinforced with Flax Fabrics Decorated with Carbon Nanostructures

**Figure 1:**
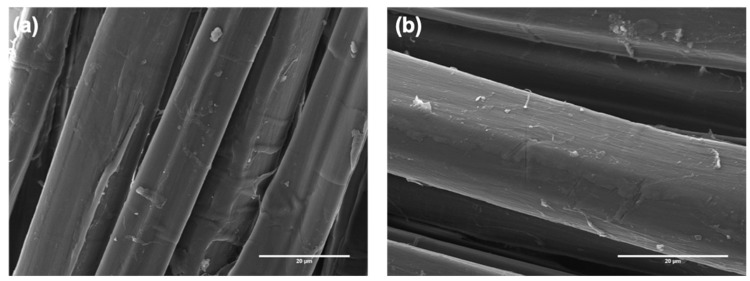
SEM micrographs showing untreated (**a**) flax yarn and (**b**) a close-up view of a single flax fibre.

**Figure 2:**
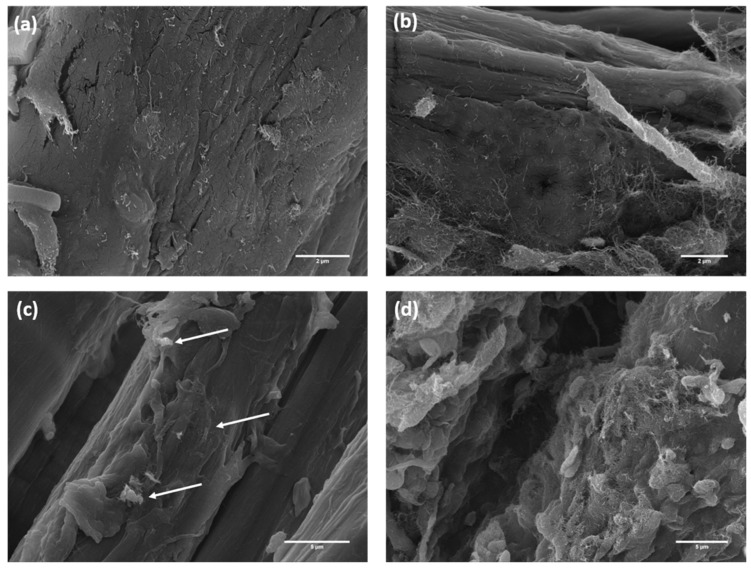
SEM micrographs showing the flax fabrics decorated with carbon nanotubes (CNTs) at different magnifications.

**Figure 3:**
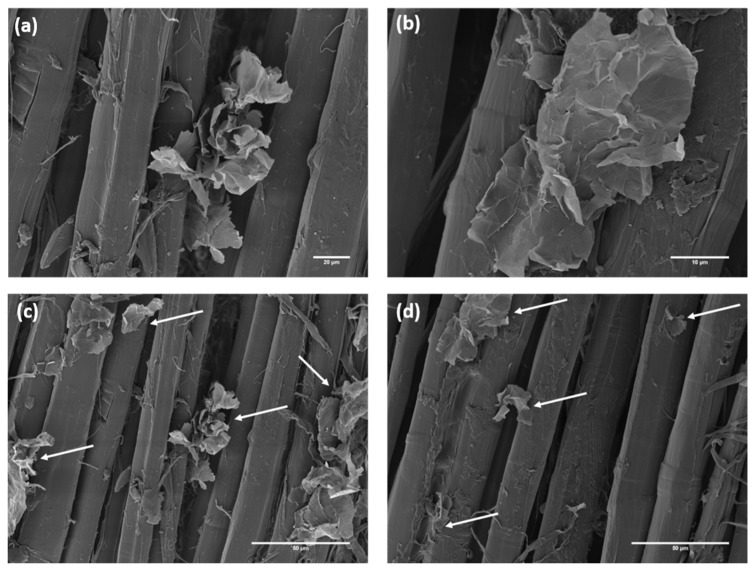
SEM micrographs showing the flax fabrics decorated with graphene nanoplatelets (GNPs) at different magnifications.

In an attempt to reduce the use of chemicals and to make the process easy and industrially scalable, a simple dip-coating method was used to decorate the flax fibers. SEM micrographs were taken to investigate the morphologies of coated and uncoated fabrics (Figure 1, Figure 2 and Figure 3). Comparing the surfaces of the untreated (Figure 1) and treated flax fabrics with CNTs (Figure 2), it is possible to observe the formation of an interconnected MWCNT network (white arrows in Figure 2c) on the fiber surface, though the dispersion was not uniform with the presence of agglomerates (Figure 2d). Untreated flax fibers (Figure 1) showed an almost smooth and featureless surface, with the presence of some impurities because no pre-treatment was applied.

The same conclusions hold for flax fabrics decorated with GNPs (Figure 3). The distribution is not completely uniform over the fiber surface, and agglomerates can be easily detected (white arrows in Figure 3c,d). These results indicate that the bonding between CNTs, GNPs, and flax fibers may only result from weak van der Waals forces with no covalent bonds. In principle, natural fibers exhibit the unique feature of having a set of hydroxyl groups in cellulose that are reactive and available for potential interactions with host nanostructures, coupled with mechanical interlocking with the rough grooves that characterize the surfaces of natural fibers. This feature was used by Sarker et al. [32] for decorating jute fibers with a uniform layer of graphene oxide (GO) by exploiting the oxygen functional groups of GO and the effects produced by an alkali pre-treatment that removed the cementing layer and exposed the hydroxyl groups of cellulose. When less-reactive graphene flakes were used, these were not fixed on the jute fiber surface. Wang et al. [31] observed a uniform deposition of CNTs on ramie fibers, but also, in this case, the fibers were subjected to an alkali pre-treatment and CNTs were prepared in a solution containing a silane coupling agent and a dispersant (polyvinylpyrrolidone). The results highlighted the positive role played by the silane coupling agent, which was essential in order to avoid agglomeration and to promote the formation of Si–O–C covalent bonds between the silane molecule and the hydroxyl groups of ramie fibers.

In the present work, to keep the number of processing steps and the amounts of chemicals at a minimum, no pre-treatments were used, and this resulted in a certain degree of agglomeration of both carbon nanostructures, which were not functionalized.

**Figure 4:**
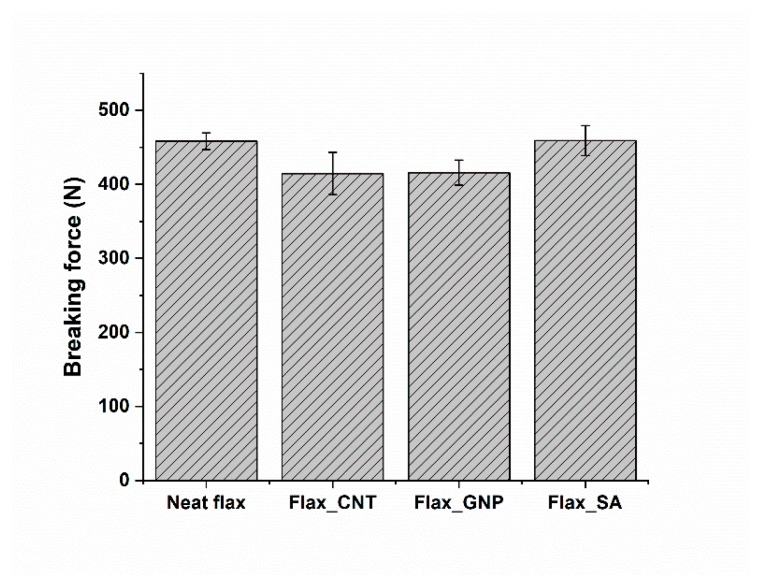
Breaking forces of untreated and surface-modified flax fabrics.

The convenient dip-coating method used did not lead to a significant reduction in the mechanical properties of the fabrics if one considers the natural variability in the mechanical responses of natural fibers, as can be inferred from the tensile tests on flax fabrics (Figure 4), thus excluding any degradation effects of the cellulose and of the cell wall materials.

GO and graphene flakes significantly increased both the tensile strength and the Young’s modulus of single jute fibers [32]. This better mechanical performance was ascribed by the authors to a kind of healing effect played by these nanostructures, in that they were able to remove stress concentrations on the fiber surfaces due to their homogeneous and uniform coating. A similar increase in tensile strength was reported in [29] on single flax fibers, again attributed to the removal of surface defects, even though, in this case, the distribution of zirconia particles was not sufficiently homogeneous.

Despite the non-optimal distribution of carbon nanostructures on the flax fabrics, the surface-modified fabrics were used for manufacturing composites based on the PP matrix. To tailor the interfacial adhesion, a standard coupling agent (MAPP) was used to explore any synergistic effects. The mechanical properties of tension and bending are summarized in Table 1, along with the percentage of variation in comparison with untreated PP/Flax composites. The incorporation of a polypropylene-grafted maleic anhydride (PPC/Flax) improved both the flexural and tensile properties due to an increased interfacial adhesion between the flax fibers and the PP matrix.

**Figure 5:**
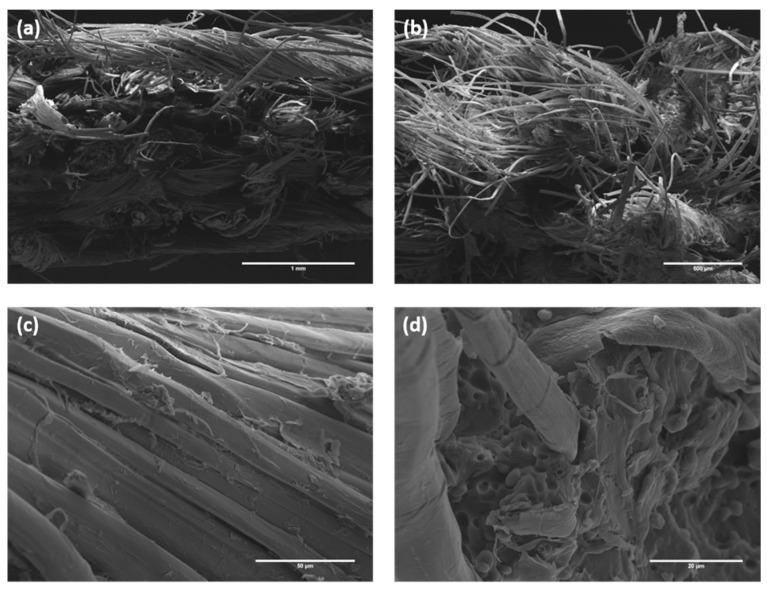
SEM micrographs of the fracture surfaces of PP/Flax composites at different magnifications.

**Figure 6:**
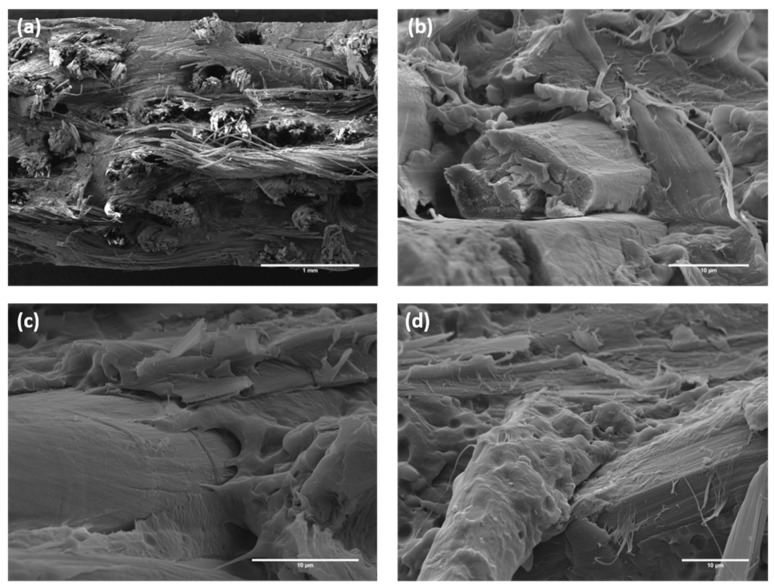
SEM micrographs of the fracture surface of PPC (PP modified with coupling agent)/Flax composites at different magnifications.

The maleic anhydride polar groups create covalent and hydrogen bonds with the flax fiber surfaces, while the polypropylene chains of MAPP form compatible blends with the bulk PP matrix through co-crystallization [37]. The improved interfacial adhesion can be readily confirmed by comparing the fracture surfaces of untreated (Figure 5) and compatibilized (Figure 6) composites.

In non-compatibilized composites, flax yarns are completely pulled out from the matrix and interfacial debonding turns out to be the dominant failure mechanism, thus suggesting a rather low fiber/matrix adhesion. Clear gaps at the interfaces between the fibers and the PP matrix can be easily observed (Figure 5d), along with the clean surfaces of the flax fibers (Figure 5c) with no matrix residues.

On the contrary, a lower degree of fiber pull-out and no gaps were found between the fibers and the matrix (Figure 6b–d), with a large amount of matrix adhering to the flax surface (Figure 6d) and fiber fractures (Figure 6b).

**Figure 7:**
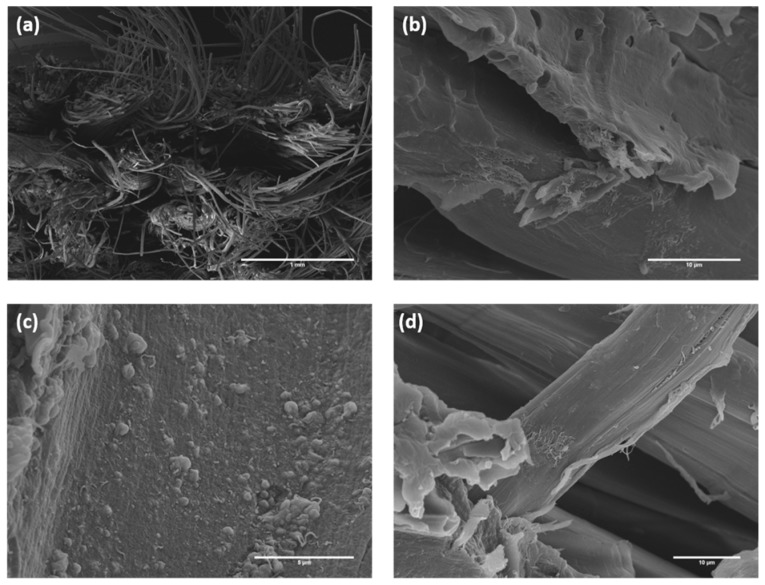
SEM micrographs of the fracture surfaces of PP/Flax_CNT composites at different magnifications.

The presence of CNTs was not beneficial, with resulting composites that exhibited reduced flexural and tensile properties to a great extent compared to untreated composites. This behavior can be ascribed to the agglomerations of CNTs which acted as stress concentrations and to their poor compatibility with the PP matrix [44,45]. Figure 7 shows the fracture surfaces of such composites, where carbon nanotubes are entangled and defects along the flax fibers can be observed (Figure 7d). The fibers are still scarcely covered by the matrix after pull-out (Figure 7b,d), and in the grooves created by the pulled-out fibers (Figure 7c), CNTs clusters can easily be seen, thus confirming the occurrence of weak van der Waals bonds between the CNTs and the flax fibers. Due to the poor mechanical results and fiber/matrix adhesion, composites with CNTs were not investigated further. In fact, it is often suggested that the existence of hydrogen bonds or other dipole–dipole interactions between maleic anhydride and modified nanotubes with carbonyl and carboxyl groups increases CNT dispersion and the properties of PP-based nanocomposites [46,47].

**Figure 8:**
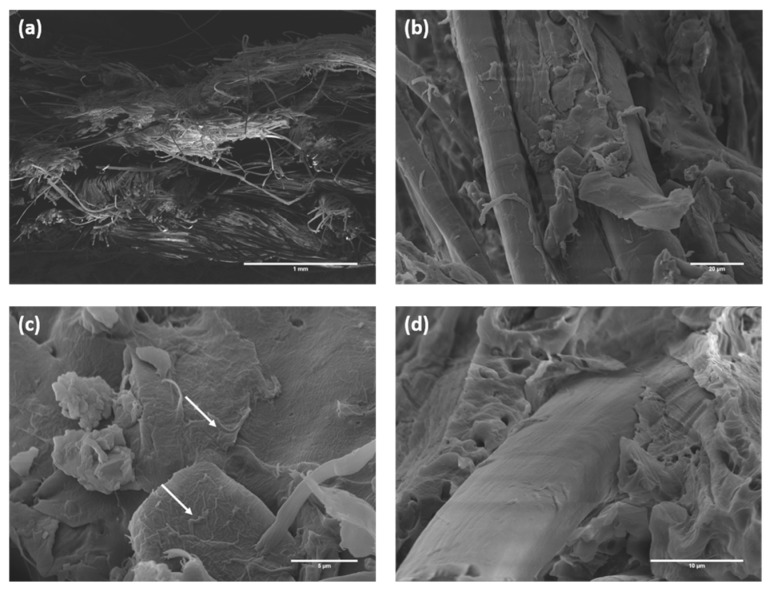
SEM micrographs of the fracture surfaces of PP/Flax_GNP composites at different magnifications.

On the other hand, the decrease in mechanical properties caused by the presence of GNPs was lower compared to CNTs. The fracture surfaces of the resulting composites (Figure 8) showed the same features as those of CNT-reinforced composites, but with a slightly better interfacial compatibility, confirmed by layers of matrix material pulled out together with the flax fibers (Figure 8b,c), where GNPs are well embedded in the polymer matrix (white arrows in Figure 8c). The relative chemical inertness of GNPs [48] did not allow the exploitation of their full potential; therefore, these composites were modified with the MAPP coupling agent.

**Figure 9:**
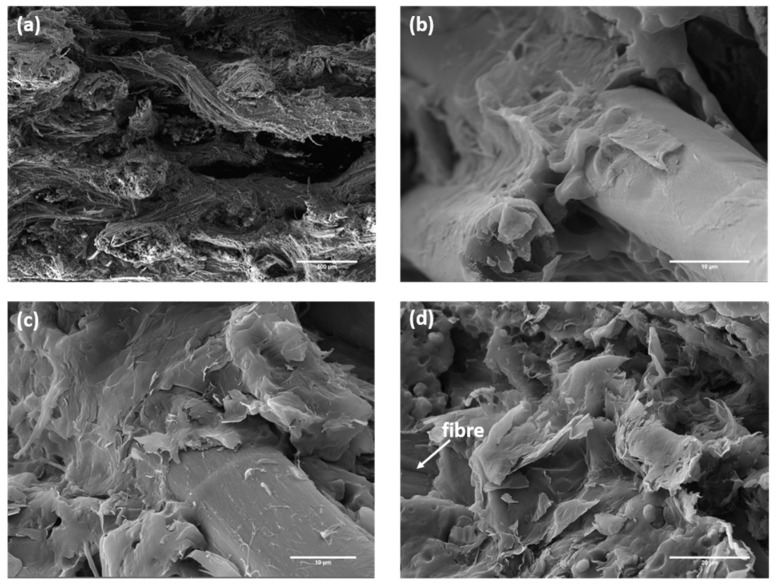
SEM micrographs of the fracture surfaces of PPC/Flax_GNP composites at different magnifications.

**Figure 10:**
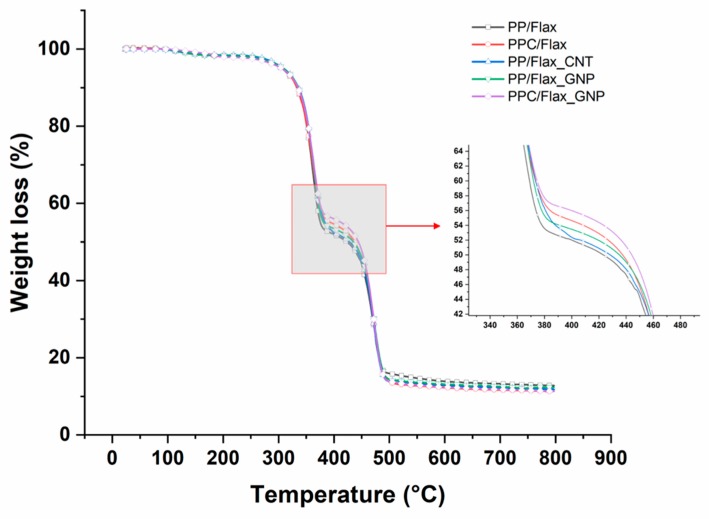
Thermograms for the different PP/flax composites.

These composites (PPC/Flax_GNP) outperformed the tensile and flexural behaviors of untreated PP/Flax composites, showing values of tensile and flexural strength even higher than those of PPC/Flax composites. As already found for GNPs in polypropylene matrices [48,49], MAPP is able to increase the chemical compatibility between PP and the polar groups of GNPs, providing a better anchoring of the GNPs into the PP matrix, which results in the enhanced adhesion between them and in a higher constraint of the polymer chains. These effects are visible in the fracture surfaces of the corresponding composites (Figure 9). In this case, it is much more difficult to differentiate the flax fibers from the PP matrix, and the extent of fiber pull-out is significantly reduced. In addition, the fibers are, in many cases, completely covered by the matrix reinforced with GNPs (Figure 9d). It is interesting to note that the tensile and flexural moduli of these composites are higher than those reported in literature for flax/PP composites [10,50,51] when considering similar fiber volume fractions, even higher than low-twisted and unidirectional MAPP-treated flax yarns in a polypropylene matrix, for which a tensile modulus of 9.26 ± 0.4 GPa was reported [52]. In addition, the tensile and flexural strength values are comparable with those of similar unidirectional composites [52]. Figure 10 shows the thermal stability (TGA) curves of the composites, and the results point out that the presence of carbon nanostructures did not markedly affect the degradation profile in comparison with untreated flax/PP composites.

While the first weight loss around 340–360 °C is due to the degradation of flax fibers and, in particular, of the cellulose [53]; the second significant weight loss (380–470 °C) is related to the degradation of PP [44]. The increased decomposition temperatures (inset of Figure 10) of the composites can be ascribed to the physical–chemical absorption of the decomposed products [54]. The physical absorption of PP molecules on the carbon nanostructures induces a delay in their volatilization, but the decomposition temperature of PPC/Flax_GNP composites is the highest among the other formulations. This significant increase cannot be due only to physical absorption, but also to a chemical absorption, thus confirming the higher level of interfacial adhesion.

### 3.2. Characterization of Composites Reinforced with Flax Fabrics Treated with Stearic Acid

Organic acids, and in particular fatty acids, are extensively used in surface treatments for particulate mineral fillers. The resulting modification causes the filler surface to become hydrophobic, thus reducing the moisture adsorption during storage and improving the incorporation of polar mineral fillers in non-polar polymer matrix melts, with reduced melt viscosity and associated enhanced dispersion [55]. These commercially available fatty acids are generally sourced from plant or animal sources and contain mixtures of mainly even-carbon-number acids. The use of stearic acid as a surface modification treatment for natural fibers has been investigated in other studies dealing with PP. This treatment is usually performed via a solution process, in which the stearic acid is dissolved in a suitable solvent, via a vapor phase [56], or by dry-blending [39]. In the first case, different solvents have been suggested in literature, including from acetone [38], toluene [57], and ethanol [58]. During the modification, it is expected that the carboxyl group of the stearic acid reacts with the hydroxyl groups of the natural fibers, but the OH groups of the different solvents, characterized by different reactivity, could also be involved. This explains why it was decided to investigate the effects of two different solvents, toluene and ethanol.

**Figure 11:**
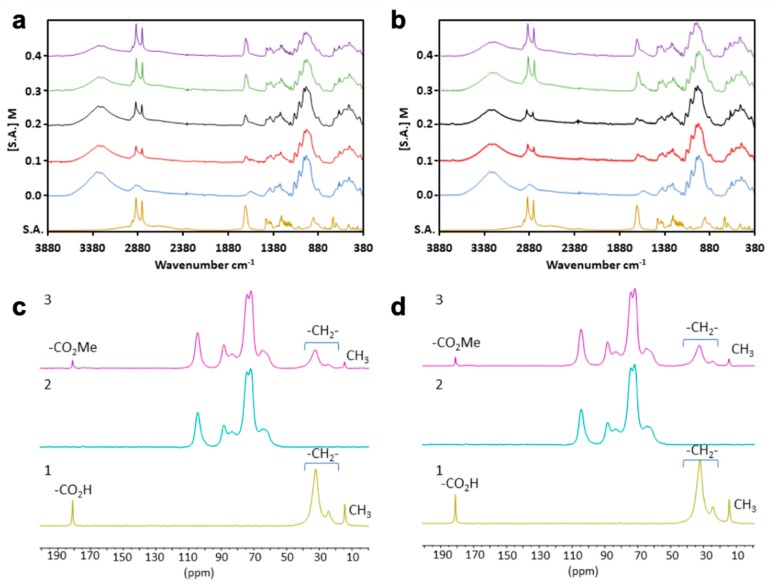
FTIR and NMR spectra for flax modified by stearic acid. (**a**) (Ethanol) and (**b**) (toluene) FTIR spectra ([SA] = 0.1, 0.2, 0.3, and 0.4 M). ^13^C CP/MAS NMR spectrum of stearic acid **1** (SA), flax fabric (blue) **2,** and, in purple, the flax fabric treated with 0.4 M of SA in ethanol (**c**) and in toluene (**d**).

At first, all of the treated flax samples were analyzed with the FT-IR and CPMAS NMR spectroscopies to understand the chemical structure. In the infrared spectra (Figure 11), the adsorption bands at about 2916 and 2848 cm^−1^ are attributed to the asymmetric (ν_as_(CH_2_)) and symmetric (ν_s_(CH_2_)) methylene vibration, while the carbonyl absorption of the carboxylic acid dimer (νC=O) for stearic acid appeared clearly at 1703 cm^−1^. This last band at 1703 cm^−1^ is a strong stretching vibrational mode of modified cellulose, which can be attributed to the ester –C=O moieties present. These are formed by esterification between –CO_2_H in stearic acid and –OH in modified cellulose, indicating that stearic acid undergoes a chemical reaction with cellulose. The main bands between 815 and 1469 cm^−1^ were attributed to the δOH, νC–O, deformation bands of (–CH_2_–)_n_, and the out-of-plane vibration bands of O–H of stearic acid dimer [59,60]. The bands at 3024–3650 cm^−1^ correspond to the stretching vibrations of the OH of cellulose, which is the main element of flax created via β1-4 linked D-glucose. The corresponding vibrational bands of C=O and OH are gradually affected as the concentration of SA increases from 0.1 to 0.4 M. This fact indicates the strong intermolecular hydrogen bond interactions between cellulose and SA. The interactions between SA and cellulose were further studied by solid-state ^13^C CP/MAS NMR (Figure 11c (ethanol) and d (toluene)) at 0.4 M. The spectrum exhibited some characteristic peaks at 181.3 and 181.0 ppm, corresponding to C from the –CO– group to free stearic acid (Figure 11c.1,d.1) and the ester group (Figure 11d.3,c.3), respectively, 104.2, 88.3, 74.4, and 71.5 ppm, corresponding to C1, C4, C5, and (C3, C2), respectively, and the peak for C6 at 64.3 ppm. These peaks could be assigned to the cellulose. In addition, 32.3 (c.3 and d.3) and 32.14 (c.1 and d.1), 24.7 and 14.5 ppm could be assigned to the aliphatic chain to SA [61,62]. The slight shift of the resonance corresponding to the group –CO– with respect to the same group –CO– of the stearic acid, together with the broadening of the signals corresponding to the –CH_2_– groups of the aliphatic chain of the stearic acid, as well as a slight chemical shift, would indicate the binding or formation of ester groups in the flax fabric after treatment. These data could indicate that, in both cases, using toluene or ethanol as solvents and at two different temperatures, the cellulose that makes up the flax fabric is functionalized with stearic acid through its OH groups.

**Figure 12:**
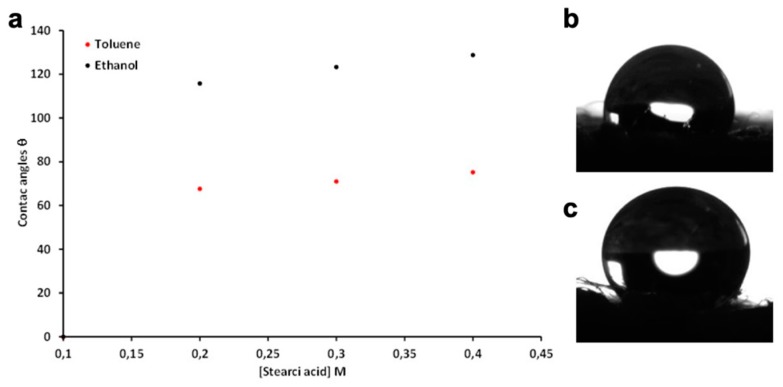
(**a**) Plot of contact angle vs. stearic acid concentration. Picture of a drop of water on flax fabric treated with 0.4 M stearic acid in toluene (**b**) or ethanol (**c**).

An assessment of the wettability of the flax fabric surfaces was performed on the flax fabrics treated with stearic acid + ethanol (0.1–0.4 M) and stearic acid + toluene (0.1–0.4 M). In Figure 12, the contact angles for the samples treated with 0.4 M of stearic acid are reported, both in toluene (b) and in ethanol (c). The images were taken from the videos at a fixed time of 10 s after water contact with the fabrics, so that all of the treated fabrics could be compared with each other. The values of the contact angles at different concentrations of stearic acid for the fabrics treated in ethanol and in toluene are reported in Table 2. 

The results shown in Table 2 demonstrate that the surface modification carried out in ethanol reaches higher values in terms of contact angle, up to 128.8° relative to a 0.4 M concentration of stearic acid, thus obtaining highly hydrophobic surfaces; likewise, it is evident that the synthesis carried out in toluene as a solvent led to lower contact angle values, leaving the surface of the flax mostly hydrophilic [63]. The differences in hydrophobicity shown in Figure 12 and Table 2 by the flax fabric samples treated in different solvents may be due to different phenomena. The reaction between an acid and an alcohol, which is known as Fischer–Speier esterification [64], produces an ester by refluxing a carboxylic acid and an alcohol in the presence of an acid catalyst. In our case, in none of the reactions was an acid used as a catalyst, but EtOH was able to yield H^+^ to the reaction media and then to act as catalyst and reaction solvent at the same time. In fact, the pKa of EtOH is 15.9, while that of toluene is 43. This fact makes EtOH a stronger acid with respect to toluene. In addition, this reaction, which takes place through cationic type intermediates, is most favored in polar media such as EtOH [65]. On the other hand, EtOH itself could give esterification processes, whereby not only SA, but also esters would be formed with the OH groups of the cellulose. This fact could significantly reduce the number of OH groups present in the sample, increasing hydrophobicity due to the loss of OH groups that can interact with water via H-bonds. Finally, the relatively high temperature at which the reaction is carried out with toluene, 100 °C, could cause some degradation of the molecular structure of the cellulose of flax, making it less reactive. The high level of hydrophobicity reached with a treatment performed in ethanol at 0.4 M allowed us to select this treatment condition for the modification of flax fabrics to be used as reinforcement in the PP matrix.

**Figure 13:**
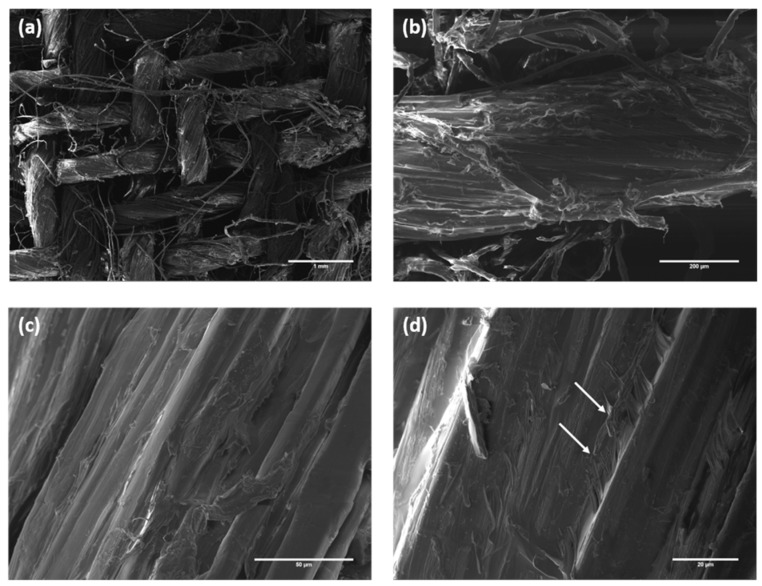
SEM micrographs showing the flax fabrics modified with 0.4 M SA at different magnifications.

To determine the structure and dispersion of stearic acid on flax fabrics before composite manufacturing, the morphologies of the resulting treated fabrics were investigated by SEM (Figure 13).

**Figure 14:**
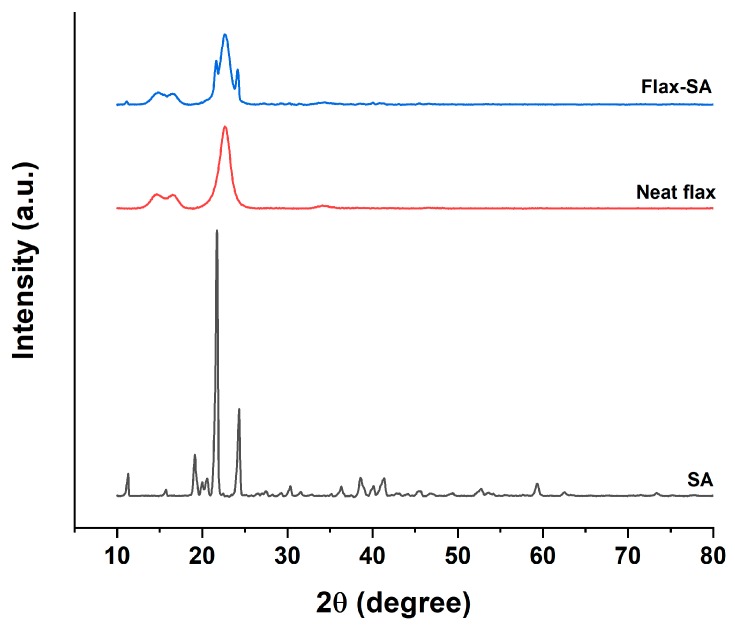
XRD patterns of pure stearic acid (SA), untreated flax fabric (Neat flax), and flax fabric modified with 0.4 M SA (Flax–SA).

The fibers appear to be covered by a thin layer of stearic acid with some micro-sized waxy protrusions (white arrows in Figure 13d), indicating a quite uniform distribution of stearic acid on the flax fiber surface. Figure 14 shows the XRD spectra of pure stearic acid and modified flax fabrics. The main characteristic peaks of the untreated flax fabric were located at 2θ = 14.7°, 16.4°, 22.6°, and 34.5°, which can be assigned to cellulose I [66], for planes $\left(1\bar{1}0\right)$, (110), (200), and (004), respectively. In the surface-modified flax fabric, additional peaks located 21.6° and 24.0° can be clearly seen, which correspond to the interplanar spacings of stearic acid, thus suggesting that the stearic acid exists in its crystal form in the modified fabric. These values can be assigned to the stearic acid monoclinic C-form, which is in line with the crystallized form obtained from solution [67].

**Figure 15:**
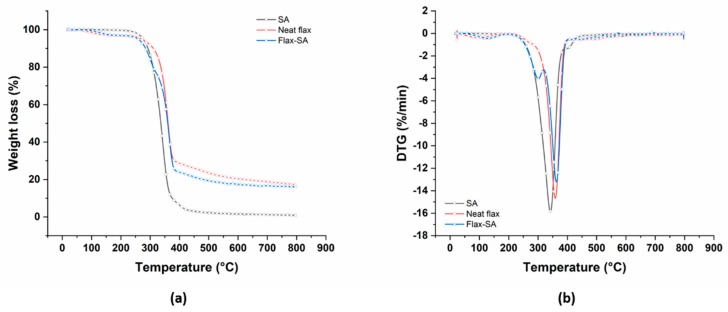
(**a**) Thermal stability (TGA) thermograms and (**b**) derivative thermogravimetry (DTG) curves of flax fabrics before and after the treatment with stearic acid (SA).

The effect of stearic acid treatment on the thermal stability of flax fabrics was investigated by thermogravimetric analysis, and the corresponding thermograms are reported in Figure 15. The thermogram of the untreated flax fabric showed the typical three peaks in the derivative curve. The first mass loss, at about 60–120 °C, is due to the release of water, a shoulder at about 240–280 °C is ascribed to the decomposition of the non-cellulosic components such as pectin and hemicellulose, and the third mass-loss peak, at about 340–360 °C, is due to the cellulose degradation [68]. A one-step mass loss of pure stearic acid was observed, with an onset weight loss temperature higher than 230 °C, which is thus compatible with the manufacturing process of PP-based composites. The thermal stability of the modified flax fabric was not significantly affected, with the exception of an additional decomposition step at a lower temperature (>230 °C) due to the vaporization of stearic acid [69], which again confirms the successful deposition of stearic acid on the flax fabric. In addition, the stearic acid treatment did not degrade the mechanical properties of the modified flax fabric, as can be inferred from the results included in Figure 4.

The reduced polar character of the modified flax fabrics resulted in composites characterized not only by higher moduli compared to composites reinforced with GNPs, but also by lower strength values, which, in any case, are higher than those exhibited by PP/Flax composites (Table 1). It is worth noting the synergistic effect on the modulus and, to a lesser extent, on the strength played by the further use of MAPP, an effect already observed by Spoljaric et al. in microcrystalline cellulose composites [57]. An improved interfacial bond strength can be the reason for this behavior. Zafeiropoulos et al. [70] reported a slight increase in stress transfer efficiency in flax fibers treated with stearic acid in the vapor phase after 36 h treatment with a PP matrix. They ascribed this improvement to the inter-entanglement of the stearic acid chains with the PP chains. The same authors also reported the development of a transcrystalline layer in stearic-acid-treated cellulose fibers [71], which was suggested to increase the interfacial adhesion, as assessed by fiber fragmentation tests. 

**Figure 16:**
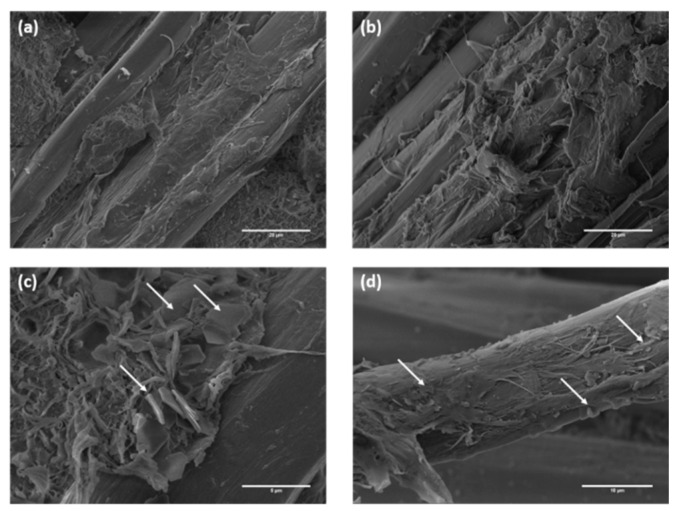
SEM micrographs of the fracture surfaces of PP/Flax_SA composites at different magnifications.

**Figure 17:**
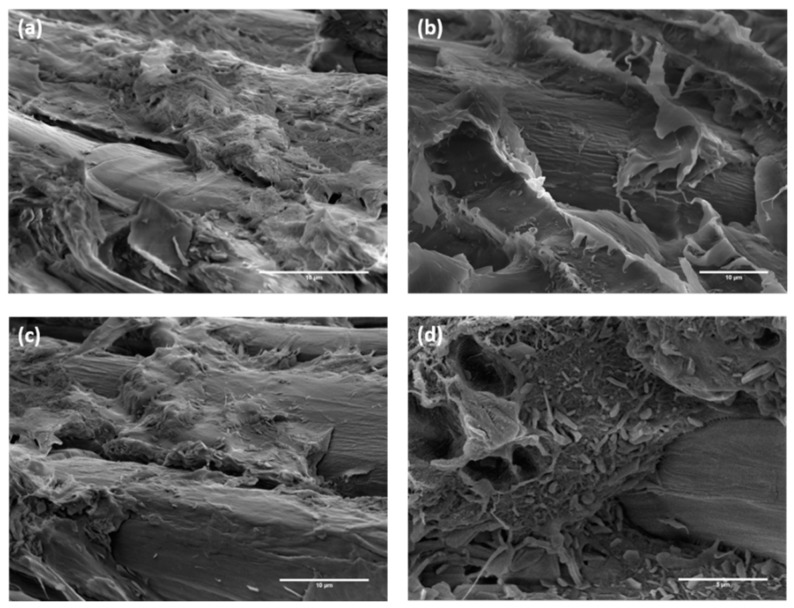
SEM micrographs of the fracture surfaces of PPC/Flax_SA composites at different magnifications.

In the present work, a better fiber/matrix adhesion was induced by the surface treatment between the hydrophobic chains of stearic acid and the polypropylene matrix and between the hydrophilic carboxyl group of the stearic acid and the flax fibers. This was confirmed by SEM analysis of the fracture surfaces of the resulting composites, reported in Figure 16 and Figure 17 for non-compatibilized (PP/Flax_SA) and compatibilized (PPC/Flax_SA) composites with stearic-acid-treated flax fibers, respectively. A strong interfacial adhesion was found in PP/Flax_SA composites, which increased after the addition of MAPP. It is possible to note the presence of a significant number of stearic acid plate-like crystals on the flax fiber surface (for instance, the white arrows in Figure 16c,d). These are supposed to create a rough surface on the flax surface, thus promoting mechanical interlocking and hindrance to polymer chain mobility, which supports the significant increase in stiffness. Flax fibers can be hardly seen in Figure 17a,b, as they are densely covered with matrix residues. The dramatic increases in the moduli were not accompanied by similar increases in strength. It is speculated that the large amount of stearic acid deposited on the fiber surface might have acted, at stresses higher than those needed to evaluate the tensile and flexural moduli, more as a lubricant than as a compatibilizer. Stearic acid is, in fact, commonly used as a processing aid to increase homogeneity and processability [39].

This consideration suggests not only the potential of stearic acid, but also the need to optimize its content in order to balance these opposing effects. 

**Figure 18:**
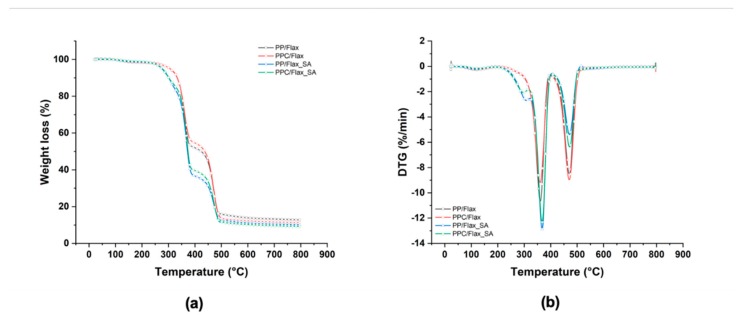
(**a**) TGA thermograms and (**b**) derivative thermogravimetry (DTG) curves of stearic-acid-treated flax fibers in a non-compatibilized (PP) or compatibilized (PPC) matrix.

Compared to untreated PP/Flax composites, the treatment with stearic acid did not modify the overall degradation profile (Figure 18), even though a lower onset temperature of thermal instability occurred in composites with stearic acid. A slight shift toward higher temperatures for the two peak mass-loss temperatures was detected after the incorporation of stearic acid. The mass-loss rate for compatibilized systems was slightly reduced, thus confirming the higher level of interfacial adhesion.

## 4. Conclusions

Four different surface modification treatments, including grafting of GNPs and CNTs, stearation, and incorporation of maleated polypropylene, were developed and applied on flax fibers to produce high-performance polypropylene-based composites. The grafting of carbon nanostructures by a simple and cost-effective dip-coating process was implemented in order to try to limit the amounts of chemicals and the number of processing steps. This resulted in a non-optimal distribution of carbon nanostructures on the fiber surface. GNPs were found to be much more effective than CNTs, leading to composites with an increased Young’s modulus and tensile strength of 54.96% and 42.46% compared to the reference ones when combined with maleated PP. These results are comparable to those obtained for unidirectional PP/Flax composites developed in other studies. The stearation treatment was optimized in terms of the solvent type and the amount of stearic acid. A 0.4 M concentration of stearic acid in ethanol provided the highest reduction in the polarity of flax fibers without altering their degradation profile and mechanical properties. The higher compatibility with apolar PP resulted in enhanced mechanical properties in tension by 102.96% and 3.39% for modulus and strength, respectively, and in bending by 61.81% and 13.97% for modulus and strength, respectively, compared to baseline composites; these were further improved by the addition of maleated polypropylene. These simple treatments, potentially prone to further optimization, can represent a step toward producing natural fiber composites with mechanical profiles compatible with semi-structural applications. 

## Figures and Tables

**Table 1:** Summary of the tensile and flexural properties of the composite materials based on polypropylene (PP) and modified flax fabrics (YM = Young’s modulus; TS = Tensile strength; FM = Flexural modulus; FS = Flexural strength; percentage variation is in reference to the corresponding property of PP/Flax).

Specimen ID	YM (GPa)	TS (MPa)	Percentage Variation, YM (%)	Percentage Variation, TS (%)	FM (GPa)	FS (MPa)	Percentage Variation, FM (%)	Percentage Variation, FS (%)
PP/Flax	6.75 ± 0.50	66.09 ± 4.83	-	-	5.42 ± 0.96	49.47 ± 6.77	-	-
PPC/Flax	9.98 ± 0.78	89.46 ± 2.33	+47.85	+35.36	7.80 ± 0.43	94.34 ± 3.68	+43.91	+90.70
PP/Flax_GNP	6.18 ± 0.17	59.54 ± 1.01	−8.44	−9.91	4.29 ± 0.25	51.25 ± 1.32	−20.85	+3.60
PPC/Flax_GNP	10.46 ± 0.29	94.15 ± 1.70	+54.96	+42.46	7.98 ± 0.32	106.36 ± 0.63	+47.23	+114.99
PP/Flax_CNT	5.17 ± 0.17	59.72 ± 3.01	−23.41	−9.64	3.69 ± 0.23	43.23 ± 2.38	−31.92	−12.61
PP/Flax_SA	13.70 ± 0.04	68.33 ± 3.73	+102.96	+3.39	8.77 ± 0.72	56.38 ± 1.11	+61.81	+13.97
PPC/Flax_SA	13.41 ± 1.69	73.28 ± 1.71	+98.67	+10.88	10.88 ± 0.88	87.44 ± 3.31	+100.74	+76.87

**Table 2:** Contact angles for flax fabrics treated with stearic acid in different solvents.

Contact angle, θ [°]		
Stearic Acid [M]	Solvent	
	Toluene	Ethanol
0.1	-	-
0.2	67.6 ± 0.05	115.8 ± 0.27
0.3	71 ± 0.32	123.3 ± 0.31
0.4	75.2 ± 0.02	128.8 ± 0.01
